# The value of poly-urethane cuffed endotracheal tubes

**DOI:** 10.1186/s13054-016-1474-3

**Published:** 2016-09-30

**Authors:** Jan Poelaert

**Affiliations:** 1 Department of Anesthesiology and Perioperative Medicine, University Hospital Brussels, Laarbeeklaan 101, 1090 Brussels, Belgium; 2 Faculty of Medicine and Pharmacy, VUB, Brussels, Belgium

I read with special interest the meta-analysis of Blot et al. in a recent issue of *Critical Care* [1]. In a systematic review of the available literature of both laboratory and clinical studies, comparing the sealing efficacy of poly-vinylchloride cuffed endotracheal tubes (PVC-ET) versus poly-urethane cuffed endotracheal tubes (PU-ET), they demonstrated a clear advantage with a demonstrated delay of chance of descent of oropharyngeal secretions when a PU-ET was used. However, prevention of ventilator-associated pneumonia could not be demonstrated in the overall data.

The characteristics of the PU cuff allow better expansion of the cuff within the trachea, in comparison with the thicker material of PVC. In a recent re-analysis of the initial data [2], we demonstrated that a clear benefit of the PU-ET is obvious after 16.6 h if patients are treated in a comparable manner with tidal volume 6 ml/kg, PEEP 7–8 cmH_2_O, and positioned in 30–40° inclined position postoperatively [3]. Furthermore, in longer term ventilated ICU patients, intubated with PU-ET, condensation fluid is sometimes present within the cuff (unpublished data), abolishing proper assessment of cuff pressure. The latter could be one of the reasons why no clear advantage is found in mechanical ventilated ICU patients. In addition, we demonstrated that, indeed, micro-aspiration still occurs with PU-ET, but is retarded for several hours [4]. This phenomenon is also suggested in in-vitro studies [5].

Another important issue which has not been discussed is the proper choice of the size of the ET. There is a clear tendency in clinical anesthesia practice to decrease the size of the ET to 8 mm internal diameter (ID) for male and 7 mm ID for female patients. This allows a theoretical improved expansion of the cuff, with a consequent decline of pleas and channels in the PVC cuffs; whether this reduction in size leads to improved sealing has still to be demonstrated.

Several open questions remain unanswered. Though there are some arguments in favor of the use of PU-ET, we still have to wait for the demonstration of a clear delay of onset of ventilator-associated pneumonia.

## Abbreviations

ET: Endotracheal tube
ID: Internal diameter
PU-ET: Poly-urethane cuffed endotracheal tube
PVC-ET: Poly-vinylchloride cuffed endotracheal tube
